# Assembled genome of an Ethiopian *Plasmodium vivax* isolate generated using GridION long-read technology

**DOI:** 10.1128/mra.00590-24

**Published:** 2024-09-16

**Authors:** Francisco Callejas-Hernández, Maria Nikulkova, Nicole Adamski, Guiyun Yan, Delenasaw Yewhalaw, Jane M. Carlton

**Affiliations:** 1 Department of Molecular Microbiology and Immunology, Johns Hopkins Malaria Research Institute, Johns Hopkins Bloomberg School of Public Health, Baltimore, Maryland, USA; 2 Department of Biology, Center for Genomics and Systems Biology, New York University, New York, New York, USA; 3 Program in Public Health, University of California, Irvine, California, USA; 4 Tropical and Infectious Diseases Research Centre, Jimma University, Jimma, Ethiopia; University of California Riverside, Riverside, California, USA

**Keywords:** *Plasmodium vivax*, genome, malaria

## Abstract

*Plasmodium vivax* causes ~20% of malaria cases in Ethiopia. Here, we report a long-read genome assembly generated from an individual collected in 2022. *P. vivax* is genetically diverse across endemic regions; thus, the genome assembly of an African isolate is an important resource for the malaria research community.

## ANNOUNCEMENT

*Plasmodium vivax* is the most widely distributed malaria parasite species and is prevalent in the Americas, Southeast Asia, and countries in Africa. In Ethiopia, ~20% of malaria cases are caused by *P. vivax* (1) although this is likely under-reported due to the failure of rapid diagnostic tests to detect low parasite densities (2). Since the first finding that *P. vivax* exhibits significantly more genetic diversity than *Plasmodium falciparum* (3), several more reports have suggested a greater capacity of *P. vivax* for functional variation (4–6). Therefore, generating representative reference genomes from *vivax* endemic regions to capture the regional diversity is essential. We report here the assembly of an African *P. vivax* isolate from an Ethiopian individual.

Blood was collected from an individual at a health center in southeast Ethiopia in October 2022. Total DNA extracted using the Qiagen Blood & Cell Culture Mini Kit was sheared using Covaris gTubes, cleaned, and size-selected using Ampure XP beads (Beckman Coulter). The sequencing library was prepared from 1 µg of DNA using Oxford Nanopore Technologies Kit V14 (SQK-LSK114), loaded onto an R10.4.1 flow cell (FLO-MIN114), and run on a GridION device for 72 hours. Basecalling of sequencing reads was performed using the MinKNOW software (version 23.07.5, Guppy version 7.0.9) with the high accuracy model (400 bp). A paired-end DNAseq library was also generated using an Illumina Nextera DNA Flex Library Preparation kit following the manufacturer’s instructions. We generated paired-end RNAseq Illumina reads (2 × 100 bp) from the same isolate. Total RNA was extracted using the Tempus Blood RNA Systems kit, and a library was prepared using the KAPA RNA Hyperprep Kit with RiboErase. Both the DNAseq and RNAseq libraries were sequenced on an Illumina Nova-seq 6000 (2 × 100 bp).

Bioinformatics software described below was used with default parameters except where otherwise noted. QC and filtering of the Illumina reads were performed using FastQC (7) and Trimmomatic (8), respectively, and reads mapping to the human genome (GRCh38), or those of low quality or containing adapter contamination, were discarded. Both sets of Illumina reads were used to polish the assembly only. Quality control of the GridION sequencing reads was performed with the MinKNOWN software (ONT version 23.07.15), and reads mapping to the human genome (GRCh38) and/or with low quality and/or containing adapter contamination were discarded. The remaining reads (*N*_50_ = 10.7 kb) were assembled using Canu version 2.2 (9) under custom parameters (increasing the correctedErrorRate). The contigs were ordered and scaffolded using RagTag (10) and the PvW1 reference sequence from Thailand, and the assembly was polished using DNAseq reads (one round, 2× coverage) and RNAseq reads (four rounds, 21× coverage) using Pilon (11). The gene content was estimated using COMPANION (12) (Pv-P01 as reference), RNAseq reads (transcriptome), and custom Python scripts. Genome completeness was calculated using BUSCO (13) (version 5.3.0) and assembly statistics using custom Python scripts.

The draft genome assembly has 14× coverage and consists of 128 scaffolds with a total size of 29.21 Mb and 39.41% G + C content. The *N*_50_ (2.1 Mb) is the second largest in the five *P. vivax* reference genomes available to date, with an *L*_50_ of only six scaffolds, similar to all the reference assemblies (Table 1). The genome completeness (96.0%) and the total gene count (4,518) were slightly lower as compared to other reference genomes, suggesting a small fraction of the genome corresponds to unresolved, fragmented, or redundant sequences in the assembly. This reference genome for an Ethiopian *P. vivax* isolate—and indeed of a *P. vivax* isolate from Africa— will be a key for comparative genomic analyses to understand the mechanisms driving genetic diversity among isolates from different regions.

**TABLE 1:** Assembly statistics of four *P. vivax* reference genomes and our new draft genome from Ethiopia

	Pv_MHC-087	Pv_Sal1	Pv_P01	Pv_W1	Pv_PAM
Country	Ethiopia	El Salvador	Indonesia	Thailand	Peru
Reference	This work	(14)	(15)	(16)	(17)
Sequencing platform	GridION	Sanger	Illumina	Illumina/PacBio	Illumina/PacBio
Total no. scaffolds	128	30^a^	242	19	28
Size (Mb)	29.21	26.8	29.04	29.00	29.43
Total gaps	216	16	422	0	0
*N*_50_ (Mb)	2.10	1.67	1.76	2.11	1.99
*L*_50_	6	6	6	6	6
*L*_90_	14	269	24	13	14
G + C content (%)	39.41	42.19	39.59	39.85	39.56
Completeness (%)	96.0	99.3	99.3	99.3	99.3
Gene count	4,518	5,530	6,523	6,075	6,412

^a^ Assigned to 14 *P*. *vivax* chromosomes; 2,745 (4.2 Mb) small contigs could not be assigned.

## Data Availability

The draft assembly (JBDKXN000000000) and sequencing reads (SRR29023276, SRR29022926, and SRR30163384) have been deposited in the NCBI sequence database under the BioProject number PRJNA1101536.

## References

[1] World Health Organization. 2023. World malaria report 2023. World Health Organization

[2] Tadesse FG, Pett H, Baidjoe A, Lanke K, Grignard L, Sutherland C, Hall T, Drakeley C, Bousema T, Mamo H. 2015. Submicroscopic carriage of Plasmodium falciparum and Plasmodium vivax in a low endemic area in Ethiopia where no parasitaemia was detected by microscopy or rapid diagnostic test. Malar J 14:303. doi:10.1186/s12936-015-0821-1 26242243 PMC4524028

[3] Neafsey DE, Galinsky K, Jiang RHY, Young L, Sykes SM, Saif S, Gujja S, Goldberg JM, Young S, Zeng Q, Chapman SB, Dash AP, Anvikar AR, Sutton PL, Birren BW, Escalante AA, Barnwell JW, Carlton JM. 2012. The malaria parasite Plasmodium vivax exhibits greater genetic diversity than Plasmodium falciparum. Nat Genet 44:1046–1050. doi:10.1038/ng.2373 22863733 PMC3432710

[4] Ford A, Kepple D, Abagero BR, Connors J, Pearson R, Auburn S, Getachew S, Ford C, Gunalan K, Miller LH, Janies DA, Rayner JC, Yan G, Yewhalaw D, Lo E. 2020. Whole genome sequencing of Plasmodium vivax isolates reveals frequent sequence and structural polymorphisms in erythrocyte binding genes. PLoS Negl Trop Dis 14:e0008234. doi:10.1371/journal.pntd.0008234 33044985 PMC7581005

[5] Arnott A, Wapling J, Mueller I, Ramsland PA, Siba PM, Reeder JC, Barry AE. 2014. Distinct patterns of diversity, population structure and evolution in the AMA1 genes of sympatric Plasmodium falciparum and Plasmodium vivax populations of Papua New Guinea from an area of similarly high transmission. Malar J 13:233. doi:10.1186/1475-2875-13-233 24930015 PMC4085730

[6] Kale S, Pande V, Singh OP, Carlton JM, Mallick PK. 2021. Genetic diversity in two leading Plasmodium vivax malaria vaccine candidates AMA1 and MSP119 at three sites in India. PLoS Negl Trop Dis 15:e0009652. doi:10.1371/journal.pntd.0009652 34370745 PMC8376102

[7] Andrews S. 2010. FastQC: a quality control tool for high throughput sequence data

[8] Bolger AM, Lohse M, Usadel B. 2014. Trimmomatic: a flexible trimmer for Illumina sequence data. Bioinformatics 30:2114–2120. doi:10.1093/bioinformatics/btu170 24695404 PMC4103590

[9] Koren S, Walenz BP, Berlin K, Miller JR, Bergman NH, Phillippy AM. 2017. Canu: scalable and accurate long-read assembly via adaptive k -mer weighting and repeat separation . Genome Res 27:722–736. doi:10.1101/gr.215087.116 28298431 PMC5411767

[10] Alonge M, Soyk S, Ramakrishnan S, Wang X, Goodwin S, Sedlazeck FJ, Lippman ZB, Schatz MC. 2019. RaGOO: fast and accurate reference-guided scaffolding of draft genomes. Genome Biol 20:224. doi:10.1186/s13059-019-1829-6 31661016 PMC6816165

[11] Walker BJ, Abeel T, Shea T, Priest M, Abouelliel A, Sakthikumar S, Cuomo CA, Zeng Q, Wortman J, Young SK, Earl AM. 2014. Pilon: an integrated tool for comprehensive microbial variant detection and genome assembly improvement. PLoS ONE 9:e112963. doi:10.1371/journal.pone.0112963 25409509 PMC4237348

[12] Haese-Hill W, Crouch K, Otto TD. 2024. Annotation and visualization of parasite, fungi and arthropod genomes with companion. Nucleic Acids Res 52:W39–W44. doi:10.1093/nar/gkae378 38752499 PMC11223846

[13] Simão FA, Waterhouse RM, Ioannidis P, Kriventseva EV, Zdobnov EM. 2015. BUSCO: assessing genome assembly and annotation completeness with single-copy orthologs. Bioinformatics 31:3210–3212. doi:10.1093/bioinformatics/btv351 26059717

[14] Carlton JM, Adams JH, Silva JC, Bidwell SL, Lorenzi H, Caler E, Crabtree J, Angiuoli SV, Merino EF, Amedeo P, et al.. 2008. Comparative genomics of the neglected human malaria parasite Plasmodium vivax. Nature 455:757–763. doi:10.1038/nature07327 18843361 PMC2651158

[15] Auburn S, Böhme U, Steinbiss S, Trimarsanto H, Hostetler J, Sanders M, Gao Q, Nosten F, Newbold CI, Berriman M, Price RN, Otto TD. 2016. A new Plasmodium vivax reference sequence with improved assembly of the subtelomeres reveals an abundance of pir genes. Wellcome Open Res 1:4. doi:10.12688/wellcomeopenres.9876.1 28008421 PMC5172418

[16] Minassian AM, Themistocleous Y, Silk SE, Barrett JR, Kemp A, Quinkert D, Nielsen CM, Edwards NJ, Rawlinson TA, Ramos Lopez F, et al.. 2021. Controlled human malaria infection with a clone of Plasmodium vivax with high-quality genome assembly. JCI Insight 6:e152465. doi:10.1172/jci.insight.152465 34609964 PMC8675201

[17] De Meulenaere K, Cuypers B, Gamboa D, Laukens K, Rosanas-Urgell A. 2023. A new Plasmodium vivax reference genome for South American isolates. BMC Genomics 24:606. doi:10.1186/s12864-023-09707-5 37821878 PMC10568799

